# Valorization of Berries’ Agro-Industrial Waste in the Development of Biodegradable Pectin-Based Films for Fresh Salmon (*Salmo salar*) Shelf-Life Monitoring

**DOI:** 10.3390/ijms23168970

**Published:** 2022-08-11

**Authors:** Janira Romero, Rui M. S. Cruz, Alexandra Díez-Méndez, Irene Albertos

**Affiliations:** 1 Faculty of Sciences and Art, Universidad Católica de Ávila (UCAV), Calle Canteros s/n, 05005 Ávila, Spain; 2 Department of Food Engineering, Institute of Engineering, Campus da Penha, Universidade do Algarve, 8005-139 Faro, Portugal; 3 MED—Mediterranean Institute for Agriculture, Environment and Development and CHANGE—Global Change and Sustainability Institute, Faculty of Sciences and Technology, Campus de Gambelas, Universidade do Algarve, 8005-139 Faro, Portugal; 4 Faculty of Health Sciences, Universidad Católica de Ávila (UCAV), Calle Canteros s/n, 05005 Ávila, Spain

**Keywords:** biopolymers, intelligent food packaging, bioactive natural compounds, biodegradable, shelf-life, sustainability

## Abstract

The healthy properties of berries are known; however, red fruits are very perishable, generating large losses in production and marketing. Nonetheless, these wastes can be revalued and used. The main objective of this study was the development of biodegradable pectin films with berry agro-industrial waste extracts to monitor salmon shelf-life. The obtained extracts from blueberries, blackberries, and raspberries wastes were evaluated in terms of flavonols, phenols and anthocyanins contents, and antioxidant capacity. Then, pectin films with the extracts of different berries were developed and characterized. The results showed that the blueberry extract film was thicker (0.248 mm), darker (L* = 61.42), and opaquer (17.71%), while the highest density (1.477 g/cm^3^) was shown by the raspberry films. The results also showed that blueberries were the best for further application due to their composition in bioactive compounds, antioxidant capacity, and color change at different pHs. The salmon samples wrapped in blueberry films showed lower values of pH and deterioration of fish during storage compared to the control and pectin samples. This study contributes to the valorization of berries agro-industrial waste by the development of eco-friendly films that can be used in the future as intelligent food packaging materials contributing to the extension of food shelf-life as a sustainable packaging alternative.

## 1. Introduction

Food packaging is an essential part of the food sector as it guarantees the quality and safety of products, helps in the transport process, provides stable storage, prevents damage and losses, and ensures greater safety for the consumer [1,2]. Traditionally, the packaging is made from petroleum derivatives; however, they generally contain chemicals considered harmful, such as bisphenol and phthalates [3]. In addition, the long life and resistance of plastics to degradation have generated an accumulation of waste that has a great negative impact on the environment [4]. For this reason, we are currently looking for plastic reduction systems and the development of sustainable alternatives. Some of these alternatives are bio-based polymers, e.g., pectin [1] that can generate biodegradable packaging materials. Likewise, these new materials can be incorporated with different compounds, improving characteristics such as permeability or optical features, which allow the production of smart packaging systems [5].

Intelligent packaging is one type of smart packaging and is defined as one capable of making decisions to increase the shelf-life of the food, inform the consumer, and improve the quality of the product through various methods such as pH indicators through color changes [6]. The containers with pH indicators may incorporate extracts of various fruits or vegetables that have different properties and allow color changes associated with the variation in the pH of the product. Some of these extracts can be obtained from berries such as blueberries, raspberries, and blackberries, which possess antioxidant capacity thanks to their bioactive compounds, such as phenolic compounds and anthocyanins [7]. Berries are a product with increasing attractiveness, and their consumption has increased in recent years due to their beneficial properties for health such as antioxidants, anticancer substances, vitamins, minerals, fibers, and other essential nutrients [8,9]. However, berries are very perishable products since they have a high water content, which makes them susceptible to mechanical damage, contamination, freezing, or dehydration, generating large losses [10]. These losses generate serious nutritional, economic, and environmental problems [11]. To circumvent these problems, the fruits are normally processed, for example, for obtaining juices, from which numerous by-products can be achieved [12]. Food waste is already a global problem that endangers the long-term food supply chain, with 1.300 million tons being discarded every year [13]. For this reason, the Sustainable Development Goals (SDGs) require, by 2030, a halving of food waste per capita in the supply and consumption chain [13]. More than 1.748 million tons of wasted food correspond to fruits and vegetables [13], so there are already several reviews on the possible applications of these residues. Bayram et al., 2021 [14] studied the possible use of biopolymers, biocomposites, smart packaging, and edible films or coatings. In the processing of fruits and vegetables, by-products consisting of seeds and shells are produced in large quantities, which present a large concentration of bioactive components such as antioxidants, pigments, proteins, essential oils, enzymes, and dietary fibers [13].

The residues resulting from processing, called pomace, also in the case of berries can be reused in the food industry as ingredients or natural additives due to the bioactive compounds [11]. As previously referred to, the waste generated represents a great loss of valuable nutrients. For this reason, the biotransformation of waste is receiving increasing attention since it can be used as a resource to obtain useful products with added value [8]. There are numerous biopolymers used in the development of packaging materials, with poly (lactic acid) (PLA), cellulose, starch, and chitosan being more widely used [15]. PLA is a biodegradable polymer with mechanical properties very similar to those of thermoplastics. Starch is also biodegradable and is a polymer that is easily found; however, it has a strong hydrophilic behavior, making it sensitive to moisture [15]. On the other hand, cellulose is one of the most abundant renewable materials being used as a filler or host polymer in packaging [15]. Chitosan contains antimicrobial properties, so it can be used as a host and antimicrobial agent [15]. As previously referred to, another polymer that can be used in the development of packaging films is pectin, in which plant extracts can be easily added to generate active ingredients [16]. Pectin is a good component thanks to its ability as a gelling and emulsifying agent, generating water-soluble films with low opacity [16]. Essential oils can be added to pectin films, the most common being cinnamon, rosemary, oregano, cloves, thyme, lemon, and orange. Other elements that can be incorporated into pectin are agricultural residues such as banana, orange, and lemon peels among others [16]. Plant extracts rich in phenolic compounds are also added to pectin to increase the antioxidant capacity of the films, although there is less research in this area compared to in essential oils [16].

On the other hand, blueberry residues have multiple uses such as biofuel, biogas, biochar, or biocrude oil production [17]. There are also various uses in food as additives; for example, Rai et al., 2021 [18] observed that blueberry residues produce certain ferments that help to improve intestinal microbiota and intestinal function, corroborating its potential use as a functional food [18,19,20]. Different studies used blueberry residues to develop smart packaging as films based on different biopolymers such as chitosan, starch, and gelatin [21,22,23,24,25,26,27]. However, no subsequent application was carried out as smart packaging on food products such as meat or fish.

Fresh fish is considered key in the diet as it provides 17% of the animal protein ingested [28]. Specifically, salmon (*Salmo salar*) is one of the most consumed products worldwide, representing 93% of production [29]. Its consumption has been increasing thanks to the fact that it contains beneficial components for health such as omega-3 long-chain polyunsaturated fatty acids (LC-PUFA) [28] as well as its color, taste, protein content, vitamins, and antioxidants [30]. However, salmon and other fishery products are very perishable due to their high-water activity, almost neutral pH, and other specific components that favor biochemical, physical, and microbial deterioration during the production chain, specifically the deterioration that begins immediately after capture [28].

To our knowledge, there is little information on the use of residues of other berries such as raspberries and blackberries for the development of smart packaging materials, as well as its comparison with blueberries and their subsequent use in fish products, particularly in salmon. In addition, few studies in the literature reported the use of pectin as a matrix to develop intelligent packaging. For this reason, this study aimed to develop and characterize biodegradable pectin films with the incorporation of blueberry, raspberry, and blackberry waste extracts and to study their effect on fresh salmon shelf-life.

## 2. Results and Discussion

### 2.1. Characterization of the Extracts

The results (Table 1) indicated significant differences (*p* < 0.05) in flavonol content among the different extracts. The blueberry extract was the one with the highest flavonols content (13.65 ± 0.01 mg of quercetin equivalents per g of extract), followed by the blackberry extract. Similar results were obtained in other studies [31,32].

Blackberry and blueberry extracts showed the highest level of anthocyanins. The raspberry extract (Table 1) presented the lowest level of anthocyanins compared with the blackberry extract (*p* < 0.05). Different studies reported significant differences between anthocyanins obtained from blackberries and raspberries, being the latter of lower content [32,33]. Sariburun et al., 2010 [32] reported that the content of anthocyanins in extracts using water was more effective as they have a high solubility in water. In a variety of raspberry called “Rubin”, anthocyanins obtained from an extract with water were 60.3 ± 0.7 mg GAE/100 g, while in an extract with methanol they were 24.1 ± 0.4 mg GAE/100 g. In the case of the blackberries, extraction with water proved to be more effective, showing values of 54.8 ± 0.8 mg GAE/100 g while with methanol the values were lower (41.3 ± 0.3 mg GAE/100 g) [32]. In short, a higher anthocyanin content was shown in blackberries, as in our study.

Blueberry extract showed the highest content of total polyphenols, differing significantly from raspberry and blackberry extracts, although their levels of polyphenols were also high. Other authors reported a higher polyphenol content in blackberry extract (24.85 ± 0.11 mg g^−1^) while that of cranberry extract was significantly lower (6.08 ± 0.04 mg g^−1^) [23] due to the extraction method in water to ensure food and environmental safety. In addition, phenols are an important indicator of antioxidant capacity [23]. In our study, the highest antioxidant capacity in both methods was obtained in blueberry extract (Table 1), while no significant differences (*p* > 0.05) were observed between the antioxidant capacity of blackberry and raspberry extracts. This shows the expected positive correlation between total polyphenol content and antioxidant capacity. The use of two different methods to measure the antioxidant capacity under different reaction conditions allowed us to establish a more precise view of the antioxidant capacity for the developed films. Gündeşli et al., 2019 [34] reported the highest antioxidant capacity in blackberries, followed by raspberries. Unlike other berries, blueberries have the greatest antioxidant capacity in the early stages of ripening, which is related to the high levels of flavonols and hydroxycinnamic acids in the pre-ripening stage [35].

On the other hand, the antioxidant capacity of raspberries was 40% lower than that of blueberries.

### 2.2. Characterization of the Developed Films

#### 2.2.1. Thickness, Density, and Hardness

The results for thickness, density, and hardness are presented in Table 2. Significant differences (*p* < 0.05) were obtained in the thicknesses of all films, with the pectin films being thinner while the blueberry films were thicker. In either case, it is shown that the addition of fruit powder significantly increased the thickness of the films compared to the control (pectin). For blueberries, similar values were recorded in the literature in the studies reported by Luchese et al., 2018 [24] and Andretta et al., 2019 [26] in cassava starch films. However, in other studies, Luchese et al., 2018 [25] and Jamróz et al., 2019 [36] obtained significantly lower thickness values, even at higher percentages of blueberry and yerba mate extracts. This decrease in thickness is possibly due to the particle size of the powder, which is within 100–300 µm. In the study by Luchese et al., 2018 [25], a lower thickness was observed in the films made from blueberry powder with smaller particle sizes. On the other hand, similar results to those obtained in the thickness of the films with blackberry extract (0.2 mm) were observed in the studies of Gutiérrez, 2018 [37] and Sganzerla et al., 2021 [38]. However, the films made with raspberry powder were thicker than those reported in Yang et al., 2021 [39], possibly due to differences in powder concentration.

Table 2 shows also the density in the different films; the film with raspberry extract was the one with the highest value (1.477 g/cm^3^) followed by the film with blueberry extract. However, the results obtained were lower than those of Yang et al., 2021 [39], possibly due to the different matrices used. In our study, the films were produced with pectin while Yang et al., 2021 [39] used a matrix comprised of pectin, sodium alginate, and xanthan gum. This could be due to the intermolecular interactions between anthocyanins and the matrix, directly influencing their properties and, therefore, the films that contain them [40], and thus, hydrogen bonds benefit from a regular arrangement of the matrix chain in the film, generating a higher density [41,42,43].

On the other hand, there was also a trend of increasing hardness from pectin films to the films incorporated with the berries’ extracts. The film with the blackberry extracts presented the highest hardness while the film with the raspberry extracts the lowest. Probably, the intermolecular hydrogen bonds were formed between the hydroxyl groups of the raspberry extract and the matrix, reducing the crosslinking of water and the matrix, thus decreasing the hardness of the film [39].

#### 2.2.2. Color

The color and opacity of the films are shown in Table 3. Significant differences were observed in all the color parameters between the different films, except for the L* parameter, where there were no significant differences (*p* > 0.05) between the film with blackberries and the film with blueberries. However, the pectin film showed the highest luminosity (L*) and hue (h*). It is worth noting a clear tendency h* decrease from pectin film (85.19) to the film with blackberries (26.19). Despite the absence of differences in the L* value between the films with blackberry and blueberry extracts, these presented different results in the other parameters. The film with blackberry extract was more reddish compared will all the other films. This may be due to the different composition of anthocyanins in the different extracts. The trend to the red/purple is the result of the presence of cyanidin, which is purple at neutral pH [23]. The results of the parameters a* and b* coincide with the ones given in the study of Kurek et al., 2018 [23], where the same trend was observed among films developed with blackberries and blueberry extracts. Of the films incorporated with berry extracts, the film with raspberry extract was the one that presented the greatest luminosity. The luminosity of the films with raspberry extract (74.75) was similar (77.76) to the raspberry films (0.5 g/L raspberry films) reported by Yang et al., 2021 [39]. 

#### 2.2.3. Opacity

The protective action against light is an important feature in the packaging of food since UV radiation and light are powerful lipid oxidizers [44]. Regarding opacity, there was a clear trend of increase in the incorporation of extracts (Table 3). The film with blueberry extract showed higher opacity than the film with blackberry extract, followed by the raspberry one.

Conversely, Kurek et al., 2018 [23] reported that blackberry extract films turned out to be opaquer than blueberry extract films.

#### 2.2.4. Color Changes at Different pH

**Figure 1:**
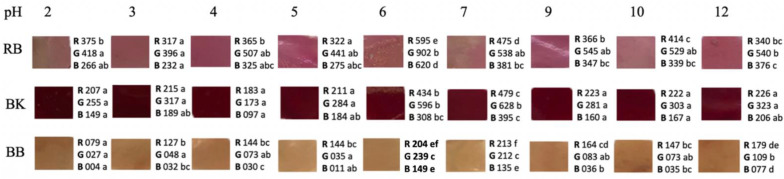
Films color changes at different pH value. RB (Raspberry), BK (Blackberry), and BB (Blueberry). Values followed by different superscript letters in the same row, for each parameter, indicate significant differences (*p* < 0.05).

The color parameters were evaluated in solutions with different pHs to determine whether color changes at an environmental pH can be shown. The highest color variability according to pH changes was presented in the films with blueberry and raspberry extracts (Figure 1).

In the case of blueberries, significant differences (*p* < 0.05) were determined in the R value from pH 2 to 12. Yellowish brown hues of pH 2–5 were obtained, turning to a slightly darker brown up to pH 12. However, the film with raspberry extract showed very few color variations at different pHs, apart from some points showing significant differences (*p* < 0.05) in R, G, and B values between pH 2 and 6. In fact, these changes were not noticeable in the visual assessment (Figure 1).

Other authors such as Kurek et al., 2018 [23] determined color parameters L, a, and b in chitosan films with blueberry and blackberry residue in a pH range of 2–12. Red colors from pH 2 to 4; blue/green at pH 5, 6, and 7; and dark green at pH 10 and 12 were observed for blueberries [23]. Separately, a red color was displayed at pH 2 and 4; violet at pH 5, 6, and 7; and dark blue at pH 10 and 12 for blackberries. In contrast, in a film of carboxymethylcellulose (CMC) and blackberries, the colors obtained in a pH range of 1 to 13 varied from pink in an acid medium to yellowish green in a basic medium [38], possibly due to the influence of the CMC matrix. Luchese et al., 2017 [22] determined the color changes in chitosan films with blueberries, on a pH scale of 2–12, obtaining results like those of Kurek et al., 2018 [23]. In addition, the process of prior blanching of the fruits caused the luminosity values to be lower, obtaining darker films [22]. This procedure was also performed in our study, which is why darker films were obtained such as those of Luchese et al., 2017 [22]. This fact is because in the production of films with unblanched fruits there is a greater degradation of anthocyanins [22]. Andretta et al., 2019 [26] observed in their study with blueberries a color variation from red/orange at acid pH and green/ yellowish at basic pH. Yun et al., 2019 [45] developed films with starch-based blueberries, obtaining colors from pale violet to intense red when exposed to hydrogen chloride. However, in exposure to ammonia, the violet turned blue and then green.

In the case of raspberry, variability was also observed, going from a yellowish rose at pH 2, 3, and 4 to a stronger rose at pH 5 and then to a yellowish rose again in the rest of the pH. In the study of Yang et al., 2021 [39], the color variation was from red to pink from pH 1 to 6, from pink to violet-blue from pH 7 to 10, and green from pH 11 to 13. These colors are because in the acidic media, the red flavylium cation form of the anthocyanins predominates, which changes its structure as the pH becomes basic until it becomes a yellow chalcone [39,40].

Therefore, the films with blueberry extract were selected in our study for the second phase of the study to monitor the shelf-life of salmon fillets due to their higher polyphenol content and second-best in anthocyanin content. In addition, the films were opaquer and showed visible color changes at different pHs.

#### 2.2.5. Biodegradation Properties

##### Soil

**Figure 2:**
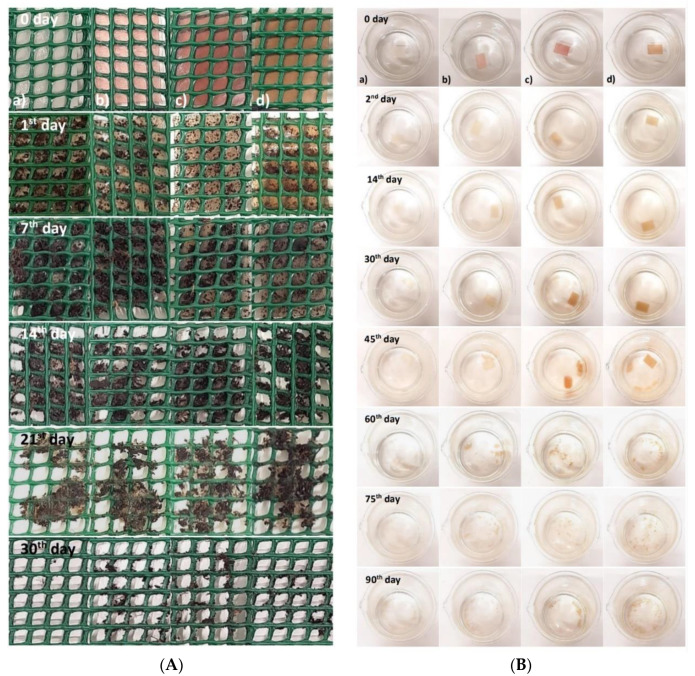
(**A**) Biodegradation test in soil; (**B**) biodegradation test in seawater: (**a**) pectin film (control), (**b**) film with raspberry extract, (**c**) film with blackberry extract, and (**d**) films with blueberry extract.

The films showed no changes in their structure after 24 h, although presented some water absorption (from the wet soil). After the seventh day, the films started to show some changes in their structure due to the solubility in water (Figure 2A).

Moreover, the organic matter and the availability of phosphorus in the soil contribute to a higher load of fungi also responsible for biodegradation [46]. A study in which cassava starch films were developed showed similar results, with signs of biodegradation after 6 days and greater changes in the degradation of the films after 12 days [47]. 

Norcino et al., 2020 [48] developed pectin-based films with copaiba oil. In this study, the films were practically biodegraded in the soil after 28 days. 

In a recent study reported by Ren et al., 2022 [49], pectin-based films also biodegraded significantly after three weeks and were biodegraded completely after five weeks.

The degradation can occur firstly from different physical and biological processes, including wetting/drying, heating/cooling, or freezing/thawing. These processes contribute to the cracking of the polymeric materials. In this study, the initial breakdown of the films occurred due to the presence of water. Then, during the degradation process, the extracellular enzymes from the microorganisms break down the polymer and the depolymerization occurs. Short chains or smaller molecules, such as oligomers, dimers, and monomers, pass the semipermeable outer bacterial membranes and are then converted to carbon dioxide, water, and biomass as the final products of the biodegradation [50,51]. The European Standard EN 13432 [52] indicates 90% as the value for packaging to be considered biodegradable by biological action in 6 months. Thus, it is possible to affirm that the developed films can be considered biodegradable.

##### Seawater

The films showed several changes during the biodegradation test in seawater (Figure 2B). After the second day, the films with the addition of berry extract kept their initial appearance but showed some loss of color. The results presented by Alvarez-Zeferino et al., 2015 [53] and Pereira et al., 2021 [54] are in agreement with the ones obtained in this study showing low levels of biodegradation in the first days of testing. 

On the 30th day, the films’ loss of color was more evident, and the seawater in which they were submerged began to show signs of clouding due to the transfer of pigments (i.e., anthocyanins) from the films to the seawater. On the 45th day, it was possible to verify that all films started to be fragmented, and the clouding of the water was also noticeable. On the 60th day, greater changes were observed in the films’ structure, as they started to fragment into small pieces and dissolve considerably in the seawater. These changes are related to the swelling of the film, as both the swelling and the solubility of the film can directly affect the water-resistance properties of the film, particularly if it occurs in a humid environment [55,56].

After 90 days, all films lost their initial rectangular shape and were quite fragmented, presenting a “flaky” appearance. Several factors influence the rate of biodegradation of the films, such as the swelling, the movement/agitation of the seawater, the existence of oxygenation, the presence of microorganisms in the seawater, and the ratio volume of seawater/film [53,56,57].

In general, biodegradation in soil was practically obtained in a short period, with an average degradation rate of 3.6% per day while the biodegradation in seawater took more time, showing an average degradation rate of 1% per day. These results are of extreme importance since they quantify and prove the fast degradation of these types of biodegradable materials against plastic or even paper packaging. Paper and plastic degradation are very slow processes, and they can take several years to be fully degraded, depending on the type of plastic or paper and the used conditions [58]. 

For example, brown newspapers started to degrade by the 10th–12th week of exposure in soil, remaining small pieces of the papers, while plastic bags had thinned off and become transparent [59]. In another study, low-density polyethylene (LDPE) bags were estimated to decompose by 50% after 4.6 years in inland (buried) and 3.4 years in marine environments [60].

### 2.3. Effect of Films Monitoring Freshness of Salmon Fillets

#### 2.3.1. pH

The results (Table 4) showed a clear upward trend in the pH for both control and pectin treatment over storage. On the other hand, the pH of the fish samples treated with the film with blueberry extract showed a tendency to be maintained until day 4 with a subsequent rise on day 7. In addition, significant differences (*p* < 0.05) were observed among the treatments from day 2. 

The initial pH values in salmon (pH > 6) for all samples with the different treatments were similar to those reported by Ambrosio et al., 2022 [28]. The increase in pH may be due to the production of ammonia and amines generated by the autolysis of nitrogenous compounds of bacteria that proliferate the decomposition process [28,61,62,63,64]. The films with the blueberry extract prevented the pH increase by avoiding the degradation of proteins and, therefore, the release of alkaline compounds during the first days of storage [65].

Therefore, the results showed a faster degradation in the control samples during the storage time than in the treated samples, especially for the film with the blueberry extract. The films composed of blueberry extract exerted a greater positive effect against deterioration since, being rich in antioxidants, these compounds can migrate to the fish surface, reducing the oxidation reactions [63,66].

#### 2.3.2. Moisture

In the first days of storage, there were no significant differences in the humidity of the samples between each treatment. From the fourth day, it is possible to observe significant differences *(p* < 0.05) between all the treatments (Table 5). A tendency of humidity decrease was also obtained throughout the storage in the treatments compared to the control samples. However, this trend only showed significance in the blueberry treatment. As in our trial, other authors obtained a reduction of humidity in the samples with the different films, possibly due to the absorption of water, which can be beneficial since it can favor the control of microbial growth [65] or due to drip loss.

#### 2.3.3. Fish Color

For the color analysis, a two-way ANOVA was performed, thus obtaining the global changes regarding the treatment and storage time.

The RGB color model is based on the mixing of red, green, and blue with different intensities to generate a color. Therefore, the color is presented as an RGB triplet. Each of the three colors can vary from zero to the maximum value (in this case 1023). The black color is represented as (000, 000, 000) and the white (1023, 1023, 1023) [67].

**Figure 3:**
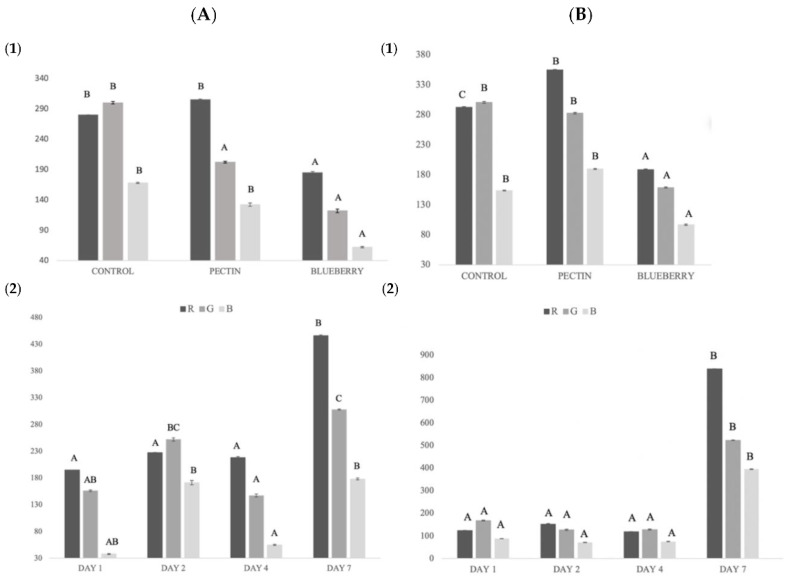
(**A**) Global salmon color changes: (**1**) global color for each treatment; (**2**) global color during storage. (**B**) Global pectin and blueberry film color changes: (**1**) global color for each treatment; (**2**) global color during storage. Different capital letters indicate significant differences (*p* < 0.05).

The color of fish is one of the most important qualities since it has a great influence on the consumer at the time of purchase [28]. Regarding the color in the salmon samples, significant differences (*p* < 0.05) were observed in all measurement parameters (R, G, and B) between the control samples and the samples with the film with blueberry extract (Figure 3).

The R value (red) obtained the highest values in the pectin treatment and on day 7 of storage. On day 7, the fish samples with a greater reddish color were observed in the samples with blueberry treatment, possibly due to the migration of the compound from the film to the fish. The same was observed in the study of Rico et al., 2020 [65], where color migration occurred from the fennel components to the sample.

On the other hand, authors such as Albertos et al., 2015 [68] obtained a significant decrease in luminosity (L*) in trout (*Trachurus trachurus*), due to the discoloration and reduction of redness during storage, as in our control samples. This reduction of reddish coloration in salmonids may be due to the oxidation of hemoproteins (hemoglobin, and myoglobin), since in their oxidized ferric form they present a brown color [68]. In the treated fish, the reddish color remained better, possibly due to the antioxidant effect of the film.

#### 2.3.4. Film Color 

Clear differences were obtained in the treatment with blueberries compared to the treatment with pectin and without any treatment (Figure 3). In the case of G, a small reduction was observed between treatments, although it was not significantly different.

Blueberry film obtained a brownish to more yellowish coloration throughout the storage time. Zhai et al., 2017 [69] showed similar results in a study to control carp freshness using colorimetric films with different anthocyanin concentrations.

The film with the lowest concentration changed from a purple color, going through green, to yellow in a period of about 6 days, while the film with the highest concentration showed no color differences until the third day. In the case of Wu et al., 2019 [70], the color changes were observed at 24 h of storage, going from purple to grayish-blue or brown depending on the anthocyanin concentration of the film.

These results were probably related to the increase of TVB-N (total volatile basic nitrogen). The antioxidant capacity of anthocyanins migrating to fish would inhibit the formation of volatile substances with nitrogen in storage [70], and their effects could vary depending on whether they are in contact with fish. In our case, the film itself simply by being on the surface of the food reduced exposure to oxygen and thus oxidation. In addition, the salmon used in our study has muscle tissue with a reddish coloration due to its high myoglobin content in comparison to Zhai et al., 2017 [69] and Wu et al., 2019 [70], which may affect the color measurement of the films covering the samples.

### 2.4. Sensorial Analysis

**Figure 4:**
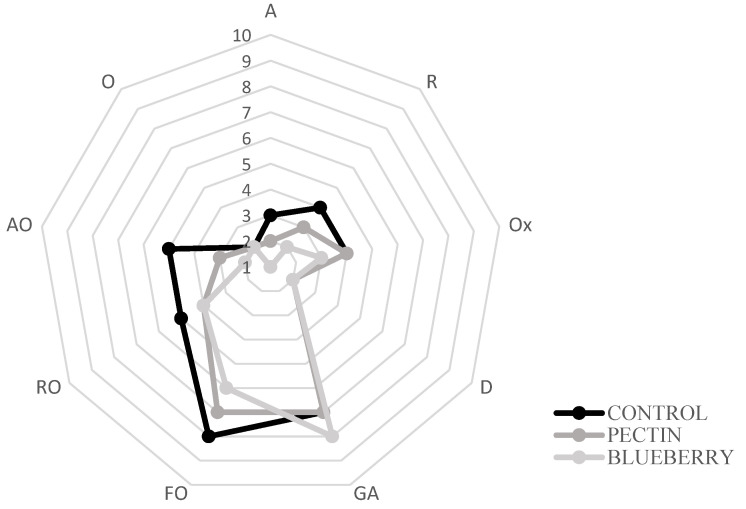
Sensorial analysis on day 2. Aromatic (A), rot (R), oxidation (Ox), dehydration (D), general acceptability (GA), fishy odor (FO), rancid odor (RO), ammonia odor (AO), and others (O).

The control samples presented the highest degree of fishy odor established by the panelists (Figure 4), while the lowest value corresponded to the treatment with the film with blueberry extracts. Albertos et al., 2015, 2018 [68,71] performed a sensory analysis in which similar results were obtained concerning the control of fish odor, rancid odor, and ammonia, which reflects the deterioration of fish meat. In addition, the introduction of red fruit extract prevented the formation of unpleasant odors (fishy smell, rancid smell, and ammonia smell) in the film-covered samples, detecting a slightly fruity aroma. The same happened in the study of Albertos et al., 2018 [71], where an “herbaceous” aroma was found in films incorporated with olive leaf powder.

The control samples showed variability in the different parameters such as fish odor, putrefactive odor, degree of dehydration, ammonia odor… etc., over storage. On the other hand, in the treatment with the film with the blueberry extract, the values remained constant. This would indicate the effectiveness of films in preserving the fish characteristics.

## 3. Materials and Methods

### 3.1. Materials

All the chemicals used in the formulation of films were food-grade quality Panreac products (Panreac Química, Barcelona, Spain). Other reagents were purchased from Sigma-Aldrich (Sigma Aldrich Chemical Co., Steinheim, Germany), and pectin was supplied by Guinama (Guinama S.L.U., Valencia, Spain).

### 3.2. Raw Materials

Blackberry, raspberry, and blueberry wastes were obtained from Viveros Campiñas (Chañe, Segovia, Spain). Berry wastes were disinfected with sodium hypochlorite (12 °C) for 15 min and immediately dried at room temperature. Afterward, blackberry, raspberry, and blueberry wastes were steamed at 100 °C for 3 min. Then, they were frozen at −83 °C until berry extract preparation. 

Gutted salmon (*Salmon salar*) was provided by Gallega de Distribuidores de Alimentación (GADISA) (Ávila, Spain). Fish was captured in the north of Galicia. The salmon was stored at 4 °C and immediately processed.

### 3.3. Development of Films with Berry Extracts

#### 3.3.1. Preparation of Berry Extracts 

Berries wastes were lyophilized (Lyoquest-55, Azbil Telstar Technologies SLU, Terrasa, Spain) and then milled and sieved (100–300 µm) to obtain the residue in powder form. 

Berry extracts were then dissolved in distilled water (12.5% *w*/*v*) for 2 h of stirring at room temperature. After, the suspension was centrifugated at 6000 rpm for 10 min and filtered through Whatman grade number 1 filter paper. The extracts were then stored at −80 °C until use.

#### 3.3.2. Development of the Films

##### Pectin Films 

**Figure 5:**
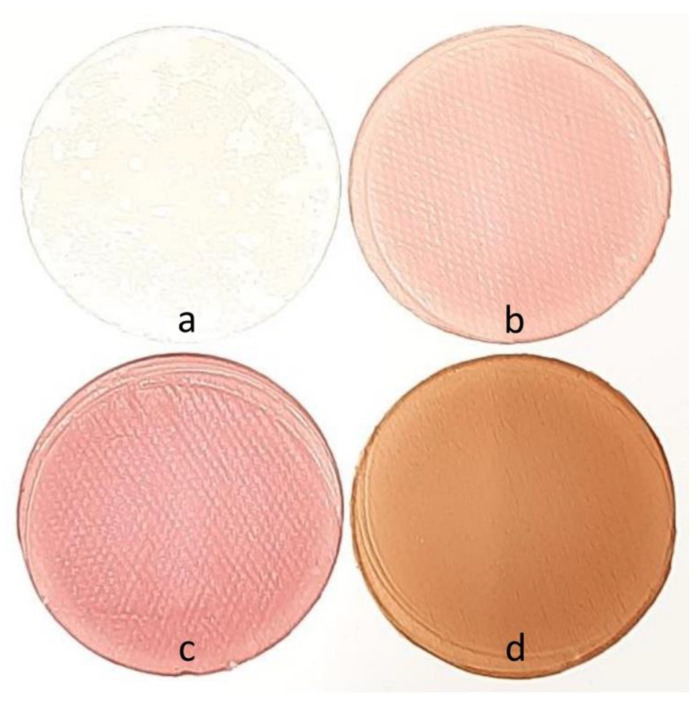
Developed films: (**a**) pectin (control); (**b**) raspberry; (**c**) blackberry; and (**d**) blueberry.

Low methoxy amidated pectin (3% *w*/*v*) was dissolved in water and stirred at 80 °C to obtain a homogenous solution. Afterward, glycerol (3%/biopolymer) was added as a plasticizer for 2 h to achieve complete dispersion. The films were obtained by casting 20 mL in 90 mm-diameter Petri dishes and dried at room temperature for 24 h. Before analyses, the films were peeled-off and conditioned in desiccators over a saturated solution of KBr (58% relative humidity) (Figure 5).

##### Films Incorporated with Berry Extracts 

Berry extracts (10% *w*/*v*) were completely dissolved in water before pectin addition. From this point, the films were prepared as previously referred (Figure 5).

### 3.4. Characterization of the Antioxidant Properties of the Extracts

#### 3.4.1. Total Phenols (TPs) Content

Total phenols were measured using the Folin–Ciocalteu method [72]. Results were expressed as mg gallic acid equivalents (GAE) per g of extract using a calibration curve with gallic acid (Sigma Aldrich Co., Steinheim, Germany) as the standard (9.8 μM to 70 μM).

#### 3.4.2. Total Flavonols Content

Total flavonols content was analyzed with Neus reagent according to Arnous, Markis, and Kefalas, 2002 [73]. Results were expressed as mg of quercetin equivalents per g of extract. 

#### 3.4.3. Total Anthocyanin Content

Total anthocyanin content was determined using the pH differential method [74]. Dilutions were prepared in 50 mL volumetric flasks with buffers pH 1.0 and pH 4.5. Absorbance was then recorded at 520 nm (Thermo Fisher Scientific, Genesys 150, Madison, WI, USA). Results were expressed as mg of malvidin-3-glucoside per L of extract. 

#### 3.4.4. DPPH (1,1-Diphenyl-2-picrylhydrazyl) Radical Scavenging Activity

The effect of antioxidant activity on DPPH was estimated according to the procedure described by Brand-Williams, Cuvelier, and Berset 1995 [75]. Results were expressed as a percentage of the inhibition of DPPH radical.

#### 3.4.5. TEAC (Trolox Equivalent Antioxidant Capacity)

The analysis was carried out according to the method reported by Re et al., 1999 [76]; 100 μL of diluted samples was mixed with 1000 μL of ABTS and working solution in an Eppendorf tube. The decay in absorbance at 734 nm was recorded over 30 min with a spectrophotometer (Thermo Fisher Scientific, Genesys 150, Madison, WI, USA). Trolox was used as the standard (7.5–240 μM). Results were corrected for moisture and expressed as μmol TE 100 g^−1^ d.m.

### 3.5. Characterization of the Developed Films

#### 3.5.1. Film Thickness and Density

The film thickness was measured using a digital micrometer (Mitutoyo, model IDC 112, Kawasaki, Japan). The results were expressed as the average of 10 replicates of samples taken from different locations on the film surface. 

Density was determined from rectangular samples of the developed films with the following dimensions: 30 mm × 20 mm. Then, each film was weighted, and for volume calculation, the thickness of each film was used. 

#### 3.5.2. Hardness

The films’ hardness (g force) was determined according to the method of Bamdad et al., 2006 [77], with some modifications. Samples were measured in six different areas, using a texturometer (Brookfield, LFRA 1500, Middleborough, MA, USA), a stainless-steel probe with 4 mm diameter (TA44), with a target value of 6 mm of penetration and a test speed of 0.5 mm/s.

#### 3.5.3. Color 

Color measurements were performed using a Minolta CR-400 colorimeter (Minolta Inc, Tokyo, Japan) with D65 as illuminant and 10° observer angle. The instrument was calibrated with a white tile standard (L* = 93.97, a* = −0.88 and b* = 1.21). To measure the color of films, a white surface was used as background. The L* parameter (lightness index scale) ranges from 0 (black) to 100 (white). The a* parameter measures the degree of red (+a) or green (−a) color and the b* parameter measures the degree of yellow (+b) or blue (−b) color. Three measurements were taken from each sample, and six samples from each film were tested. 

#### 3.5.4. Opacity

The opacity of the samples was calculated based on the method reported by Martins et al., 2010 [78], as the relationship between the opacity of each sample in a black standard (Yb) and the opacity of each sample in a white standard (Yw), as can be seen in Equation (1):

Opacity (%) = Yb/Yw × 100(1)

#### 3.5.5. Color Changes at Different pHs

The films were cut into pieces of 2 cm^2^ and were immersed in different pH buffers to adjust pH values to 2, 3, 4, 5, 6, 7, 8, 9, 10, and 12. Afterward, the color was determined with a colorimeter on solids PCE-RGB (PCE Ibérica S.L., Albacete, Spain).

### 3.6. Biodegradation Tests

#### 3.6.1. Soil

The biodegradation test in soil was based on the methodology used by Pereira et al., 2021 [54]. The films were cut (3 cm × 2 cm) and placed inside a perforated polyethylene net (5 cm × 4 cm; mesh opening 4 mm). Then, the films were buried in soil (Eco grow: nitrogen = 80–150 mg L^−1^; phosphorus = 80–150 mg L^−1^; potassium = 80–150 mg L^−1^; organic Matter = >70%; pH = 5.5–6.5; humidity = 50–60%; conductivity = 0.2–1.2 EC) at a distance of 11 cm from the surface in a rectangular vase (71 cm × 26 cm × 25.5 cm) and with a distance of 5 cm between each film. The soil was watered with 500 mL of water every 7 days at 25 °C. The films’ appearance was photographed, and the area of biodegradation was measured during the time of the experiment. This test was carried out in triplicate for each sample.

#### 3.6.2. Seawater

The biodegradation test in seawater was based on the methodology used by Pereira et al., 2021 [54]. The films were cut (3 cm × 2 cm) and submerged in 300 mL of seawater (Faro, Portugal, pH = 7.20). The samples were shaken at 150 rpm (Edmund Bühler, KL2 shaker, Tübingen, Germany) and 25 °C. The films’ appearance was photographed during the time of the experiment. This test was carried out in triplicate for each sample.

### 3.7. Monitoring the Shelf-Life of Salmon Fillets

#### 3.7.1. Preparation and Treatments of Salmon Samples

Gutted salmon (*Salmon salar*) was skinned and filleted, then cut into pieces of 5.7 × 2.5 cm (weighing approximately 10 g) and randomly allocated into 3 batches: fish without film (control), pectin film (pectin), and pectin film with blueberry extract. Each fish sample was individually wrapped with 90 mm-diameter films (control, pectin, and blueberry) with the help of tweezers under hygienic conditions. Afterward, all treatments were stored at 4 °C for 7 days. The assay was run in duplicate. All analyses were performed in triplicate.

#### 3.7.2. pH

Each fish sample (10 g) was homogenized in 100 mL of distilled water, and the mixture was filtered. The pH (pH-meter model basic 20, Crison, Barcelona, Spain) of the filtrate was measured at room temperature. 

#### 3.7.3. Moisture

The moisture content of each sample was gravimetrically determined [79]: 10 g of fish were prepared until constant weight in an air oven at 100 °C for about 24 h. The moisture was expressed in percentage.

#### 3.7.4. Color Changes

Color changes of films and fish surface without film were measured using a colorimeter on solids PCE-RGB (PCE Ibérica S.L., Albacete, Spain) in the same conditions as previously referred to in Section 3.5.5.

#### 3.7.5. Sensorial Analysis

Samples were subjected to a descriptive test. A trained panel consisting of 10 panelists, formed by 5 men and 5 women, with ages within 25–30, were recruited from the Catholic University of Ávila, for their previous experience in sensory analysis.

In the descriptive test, the panelist scored the following different attributes: fishy (off-odors, putrefaction) odor intensity, aromatic odor intensity, ammonia odor intensity, other odors, drip loss, color without film (oxidation), and general acceptability. The scores ranged between 1 and 10.

### 3.8. Statistical Analysis

Data were analyzed by a one-way ANOVA. Fisher’s LSD (Least Significant Difference) test was applied at a significance level of 0.05 for determining group differences. In the color changes of salmon fillets, a two-way ANOVA (treatment, time) was performed. Kruskal–Wallis test was used to examine differences in the sensorial analysis. The software Statgraphics Centurion XVI was employed for carrying out the statistical analysis.

## 4. Conclusions

Berries are a source of anthocyanins and can be used as an effective method for controlling the quality and freshness of food as pH indicators. The concentration of anthocyanins in the developed films was a key factor influenced by both the intensity and the rate of color change. The addition of berry extracts to pectin films contributed to changing the films’ properties. Moreover, the pectin films’ supplement with blueberry extracts not only showed biodegradability properties in soil and seawater but also protected the salmon samples from deterioration due to their anthocyanin content and antioxidant capacity, increasing the salmon’s shelf-life. This study contributes to the valorization of berries’ agro-industrial waste by the development of eco-friendly films that can be used in the future as food packaging materials to meet the market demands. However, more studies are needed to evaluate different microbiological and quality parameters.

## Figures and Tables

**Table 1:** Characterization of the antioxidant properties of the extracts.

	Total Polyphenols	Total Flavonols	Total Anthocyanins	DPPH	ABTS-TEAC
	(mg GAE/100 g)	(mg of Quercetin Equivalents per g of Extract)	(mg of Malvidin-3-Glucoside per L of Extract)	(% Inhibition)	(µmol Trolox/g ext.)
**Raspberry**	11.80 ± 0.060 ^a^	0.99 ± 0.010 ^c^	3.54 ± 0.500 ^a^	20.65 ± 1.340 ^a^	1.63 ± 0.110 ^a^
**Blackberry**	11.80 ± 0.050 ^a^	3.90 ± 0.990 ^a^	13.21 ± 2.710 ^b^	20.85 ± 1.201 ^a^	1.48 ± 0.180 ^a^
**Blueberry**	12.14 ± 0.080 ^b^	13.65 ± 0.010 ^b^	8.58 ± 0.870 ^ab^	34.00 ± 0.282 ^b^	2.67 ± 0.135 ^b^

The values (mean ± standard deviation) followed by different lowercase letters in the same column, for each parameter, are significantly different (*p* < 0.05).

**Table 2:** Thickness, density, and hardness of the developed films.

	Thickness (mm)	Density (g/cm^3^)	Hardness (g Force)
**Pectin**	0.128 ± 0.009 ^a^	1.124 ± 0.023 ^a^	495.267 ± 15.482 ^a^
**Raspberry**	0.174 ± 0.044 ^b^	1.477 ± 0.035 ^b^	863.756 ± 12.142 ^b^
**Blackberry**	0.200 ± 0.008 ^c^	1.316 ± 0.015 ^c^	882.467 ± 7.688 ^c^
**Blueberry**	0.248 ± 0.009 ^d^	1.380 ± 0.007 ^d^	763.156 ± 10. 650 ^d^

The values (mean ± standard deviation) followed by different superscript letters in the same column, for each parameter, are significantly different (*p* < 0.05).

**Table 3:** Color and opacity of the developed films.

	L*	a*	b*	C*	h*	Opacity (%)
**Pectin**	91.377 ± 0.437 ^c^	1.104 ± 0.164 ^a^	13.076 ± 0.712 ^a^	13.126 ± 0.721 ^a^	85.194 ± 0.549 ^d^	11.373 ± 0.193 ^a^
**Raspberry**	74.753 ± 0.563 ^b^	23.678 ± 0.225 ^c^	14.620 ± 0.351 ^c^	28.056 ± 1.071 ^b^	31.687 ± 0.462 ^b^	13.028 ± 0.106 ^b^
**Blackberry**	61.102 ± 1.722 ^a^	28.423 ± 1.111 ^d^	13.976 ± 0.378 ^b^	31.672 ± 1.159 ^d^	26.188 ± 0.355 ^a^	16.961 ± 0.299 ^c^
**Blueberry**	61.422 ± 1.378 ^a^	16.04 ± 0.537 ^b^	25.431 ± 0.191 ^d^	30.070 ± 0.344 ^c^	57.766 ± 0.862 ^c^	17.71 ± 0.190 ^d^

The values (mean ± standard deviation) followed by different superscript letters in the same column, for each parameter, indicate significant differences (*p* < 0.05).

**Table 4:** pHs of salmon fillets during storage with different treatments.

	pH			
	Day 1	Day 2	Day 4	Day 7
**Control**	6.57 ± 0.021 ^a B^	6.40 ± 0.030 ^c A^	6.97 ± 0.052 ^c C^	7.20 ± 0.020 ^b D^
**Pectin**	6.40 ± 0.000 ^a C^	6.24 ± 0.011 ^b B^	6.97 ± 0.021 ^b A^	6.92 ± 0.020 ^a D^
**Blueberry**	6.24 ± 0.010 ^a D^	6.00 ± 0.000 ^a C^	5.58 ± 0.013 ^a B^	6.95 ± 0.000 ^a A^

The values followed by different lowercase superscript letters in the same column, for each parameter, are significantly different (*p* < 0.05). The values followed by different capital superscript letters in the same row, for each parameter, are significantly different (*p* < 0.05).

**Table 5:** Moisture (%) of salmon fillets during storage with different treatments.

	Moisture (%)			
	Day 1	Day 2	Day 4	Day 7
**Control**	63. 48 ± 0.011 ^a A^	65.29 ± 0.030 ^a A^	68.43 ± 0.010 ^c A^	67.32 ± 0.010 ^b A^
**Pectin**	63.48 ± 0.020 ^a B^	55.72 ± 0.012 ^a A^	54.46 ± 0.030 ^b A^	51.77± 0.021 ^a A^
**Blueberry**	63.48 ± 0.000 ^a C^	57.96 ± 0.011 ^a BC^	44.11 ± 0.000 ^a A^	54.28± 0.010 ^a B^

The values followed by different lowercase superscript letters in the same column, for each parameter, are significantly different (*p* < 0.05). The values followed by different capital superscript letters in the same row, for each parameter, are significantly different (*p* < 0.05).
